# Test your knowledge and understanding

**Published:** 2017-05-12

**This page is designed to help you to test your own understanding of the concepts covered in this issue, and to reflect on what you have learnt.**

**Figure:**
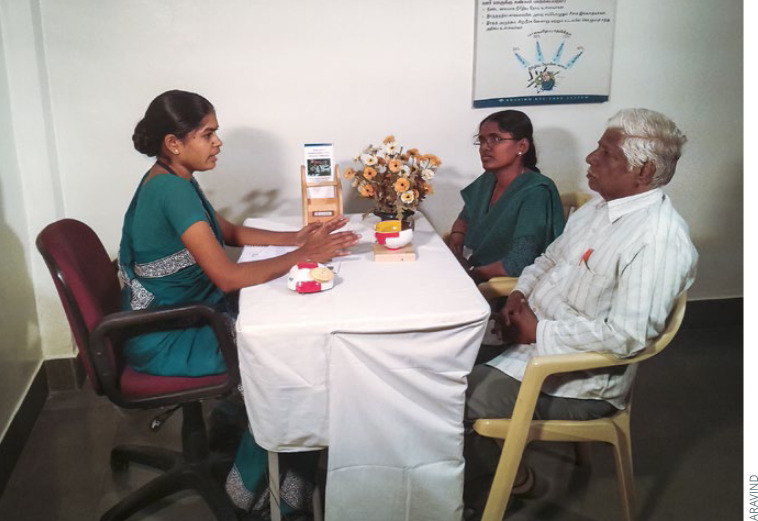
Communication skills are an important component of CPD. INDIA

We hope that you will also discuss the questions with your colleagues and other members of the eye care team, perhaps in a journal club. To complete the activities online – and get instant feedback – please visit **www.cehjournal.org**

**Tick ALL that are TRUE**

**Question 1 Continuing Professional Development:**
  □ **a.** Is more relevant for doctors than nurses
  □ **b.** Should only be necessary after a clinical incident
  □ **c.** Should be driven by patients
  □ **d.** Must always be followed by an assessment
  □ **e.** Applies only to registered eye care practitioners
**Question 2 We should continue to learn:**
  □ **a.** In order to remain on the register
  □ **b.** To ensure the best outcomes for our patients
  □ **c.** To avoid litigation
  □ **d.** To address the gaps in our knowledge
  □ **e.** To maximise our income
**Question 3 The following are relevant ways of maintaining CPD:**
  □ **a.** Discussing a difficult case with a colleague
  □ **b.** Attending a journal club
  □ **c.** Going to a product promotional meeting
  □ **d.** Participating in a routine ward round
  □ **e.** Attending equality and diversity training
**Question 4 Which of the following are untrue about CPD:**
  □ **a.** Institutional buy-in is not necessary for a CPD programme
  □ **b.** Leadership training only applies to senior staff
  □ **c.** It is best to plan CPD at least 5 years in advance
  □ **d.** Effective CPD requires learning from a more senior person
  □ **e.** Communication skills are most relevant for HR personnel

## ANSWERS

All are false. CPD is relevant for everyone. It should not be a knee-jerk reaction to an ‘incident’. CPD should be driven within the profession but encouraged by patients. An assessment is not always necessary but reflection on the learning is helpful. CPD applies to all the eye health care team.
b) and d) are correct. Motivation for CPD should be related to improving knowledge and outcomes rather than just remaining on the register, avoiding litigation or making money.
All are relevant but not all may be recognised for gaining CPD ‘points’, for example attending a product promotion or attending a routine ward round.
All are false. The institution should always be involved and supportive of the CPD programme. Leadership training is relevant for all members of the team. Planning CPD too far ahead is a mistake as circumstances may change. We can all learn from each other, whether more senior or junior. Communication skills are vital for everybody – not just the human resource department!

